# Oncogenic roles of TOPK and MELK, and effective growth suppression by small molecular inhibitors in kidney cancer cells

**DOI:** 10.18632/oncotarget.7755

**Published:** 2016-02-26

**Authors:** Taigo Kato, Hiroyuki Inoue, Seiya Imoto, Yoshinori Tamada, Takashi Miyamoto, Yo Matsuo, Yusuke Nakamura, Jae-Hyun Park

**Affiliations:** ^1^ Department of Medicine, The University of Chicago, Chicago, IL, USA; ^2^ Human Genome Center, Institute of Medical Science, The University of Tokyo, Tokyo, Japan; ^3^ OncoTherapy Science Inc., Kawasaki, Japan; ^4^ Department of Surgery, The University of Chicago, Chicago, IL, USA

**Keywords:** kidney cancer, TOPK, MELK, molecular target, kinase inhibitor

## Abstract

T–lymphokine-activated killer cell–originated protein kinase (TOPK) and maternal embryonic leucine zipper kinase (MELK) have been reported to play critical roles in cancer cell proliferation and maintenance of stemness. In this study, we investigated possible roles of TOPK and MELK in kidney cancer cells and found their growth promotive effect as well as some feedback mechanism between these two molecules. Interestingly, the blockade of either of these two kinases effectively caused downregulation of forkhead box protein M1 (FOXM1) activity which is known as an oncogenic transcriptional factor in various types of cancer cells. Small molecular compound inhibitors against TOPK (OTS514) and MELK (OTS167) effectively suppressed the kidney cancer cell growth, and the combination of these two compounds additively worked and showed the very strong growth suppressive effect on kidney cancer cells. Collectively, our results suggest that both TOPK and MELK are promising molecular targets for kidney cancer treatment and that dual blockade of OTS514 and OTS167 may bring additive anti-tumor effects with low risk of side effects.

## INTRODUCTION

Kidney cancer is the seventh most common cancer and the tenth most common cause of cancer death in men, and it is also the tenth most common cause of cancer in women [1]. In 2015, the number of new kidney and renal pelvis cancer cases was estimated to be 61,560 that led to more than 14,080 deaths in United States [1]. The 5-year disease-specific survival has improved from about 50% in 1975-1977 to 65% in 2000-2005, although that of patients at an advanced stage still remains poorly with around 10% rate [2–4]. 30% of patients who underwent surgery of localized kidney cancer develop distant metastasis and have limited therapeutic options, such as tyrosine kinase inhibitor (TKI) and mammalian target of rapamycin (mTOR) inhibitors [5]. In addition, the clinical effects of these drugs are very limited and patients often discontinue administration of these drugs due to severe side effects including hand-foot syndrome, liver dysfunction and interstitial pneumonia [6–10]. Therefore, development of more effective therapy for kidney cancer is eagerly expected.

TOPK (T-lymphokine-activated killer cell-originated protein kinase, also known as PBK or PDZ-binding kinase) is a Ser/Thr protein kinase that is highly expressed in various types of human cancer [11–14]. TOPK is known to be activated during the cell mitosis process and play a critical role in cytokinesis. We previously reported that knockdown of TOPK caused dysfunction of cytokinesis and subsequently apoptosis of cancer cells [13].

MELK (maternal embryonic leucine zipper kinase, also known as MPK38 or murine protein serine/threonine kinase 38) is a cell-cycle dependent protein kinase that belongs to the AMP-activated Ser/Thr protein kinase family [15, 16]. Elevated expression of the *MELK* gene is correlated with poorly differentiated histological types of brain tumor and prostate cancer [17, 18], and with poor prognosis of breast cancer patients [19].

The two molecules, TOPK and MELK, have shown similar expression patterns; they are up-regulated in various types of cancer including cancer stem cell-enriched tumors and more importantly their expressions are hardly detectable in normal organs except in the testis [11, 20]. Moreover, MELK expression levels were strongly correlated with those of forkhead box protein M1 (FOXM1) known as an important transcriptional factor and a master regulator of mitosis in cancer stem cells [21, 22]. These results suggest a possible close link among TOPK, MELK, and FOXM1 in a growth regulation pathway in cancer cells, which may provide a new strategy for successful treatment of cancer patients. Hence, we have developed TOPK inhibitors (OTS514 and OTS964) and a MELK inhibitor (OTS167) that showed therapeutic potentials in pre-clinical models of human cancer [23, 24].

In the present study, we demonstrate that TOPK regulates FOXM1 like as MELK does and that knockdown of either TOPK or MELK effectively suppresses the growth signaling pathway composed of these three oncoproteins. We also demonstrated that the combination of OTS514 and OTS167 can effectively reduce the expression levels of TOPK, MELK and FOXM1, and decreased viability of kidney cancer cells. These findings suggest that dual blockade using a combination of a TOPK inhibitor (OTS514) and a MELK inhibitor (OTS167) at the lower dose may be a promising molecular-targeted therapy for kidney cancer patients with avoidance or reduction of their toxicity.

## RESULTS

### TOPK and MELK expression in kidney cancer cell lines

**Figure 1:**
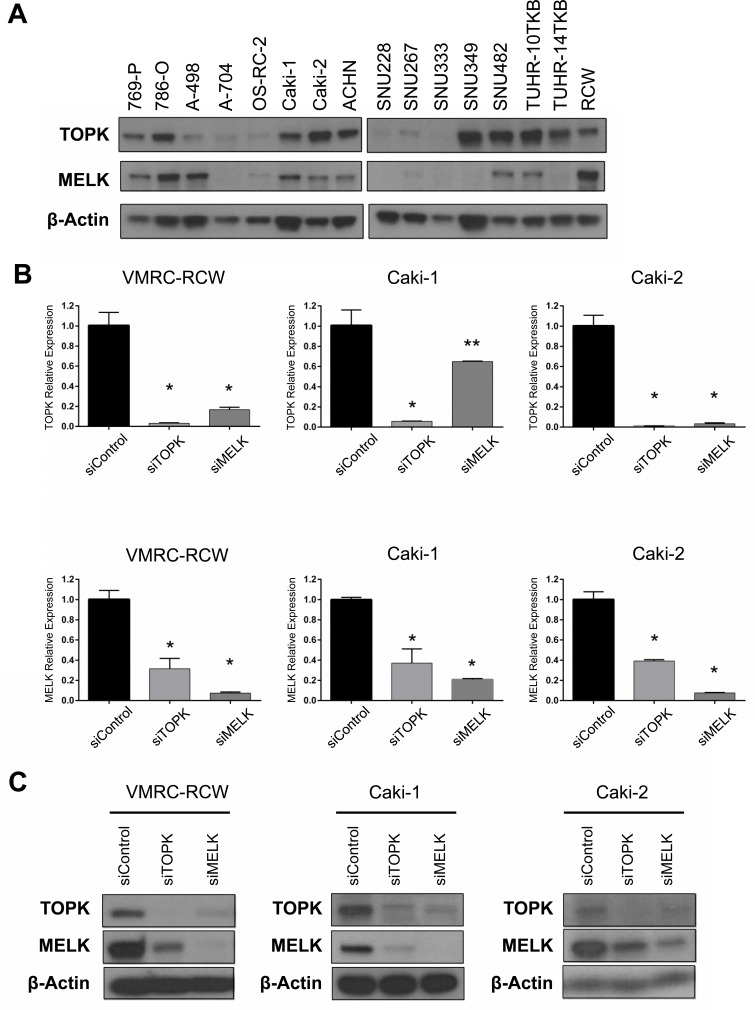
Expression and knockdown effects of TOPK and MELK in kidney cancer cell lines **A.** Expression of endogenous TOPK and MELK protein in 16 kidney cancer cell lines examined by Western blot analysis. **B.** The transcriptional level of *MELK* was downregulated by TOPK knockdown with siTOPK. MELK knockdown also led to downregulation of *TOPK* in the transcriptional level. **C.** Silencing of TOPK expression with siTOPK also reduced the MELK expression in kidney cancer cell lines. TOPK expression was also suppressed by MELK knockdown with siMELK. *p < 0.01, **p < 0.05 compared with the corresponding value of the siControl group.

We examined expression levels of *TOPK* and *MELK* genes in kidney cancers through publically-available gene expression datasets. The Oncomine database revealed that both *TOPK* and *MELK* genes are significantly up-regulated in kidney cancers (Supplementary Figure S1). Interestingly, the Cancer Genome Atlas (TCGA) data showed that expression levels of *TOPK* and *MELK* are strongly correlated in various cancer types as shown in Supplementary Figure S2, suggesting that *TOPK* and *MELK* may be regulated by a common transcription pathway or may be in some positive feedback loop [25–27]. Based on these findings, we investigated expression levels of TOPK and MELK in 16 kidney cancer cell lines by western blot analysis (Figure 1A). Although some cell lines showed the discordance in TOPK and MELK protein levels, most of the cell lines examined revealed the concordant expression levels, further suggesting some interaction between TOPK and MELK.

### Knockdown effects of endogenous TOPK and MELK

To investigate the biological function of TOPK and MELK in kidney cancer cells, we used siRNA (small interfering RNA) to knockdown TOPK and MELK expression using three kidney cancer cell lines, VMRC-RCW, Caki-1, and Caki-2 in which TOPK and MELK were highly co-expressed (Figure 1A). Each of siRNA successfully knocked down the transcript levels of the target genes (Figure 1B) and also significantly reduced the amount of its target protein (Figure 1C). However, unexpectedly, knockdown of TOPK led to reduction of MELK protein level and *vice versa* knockdown of MELK reduced TOPK protein level (Figure 1C). The semi-quantitative RT-PCR revealed that the expression of *MELK* was also downregulated by TOPK knockdown and *vice versa* knockdown of MELK downregulated *TOPK* transcription level (Figure 1B), suggesting that TOPK and MELK are likely to be influenced each other.

### TOPK and MELK knockdown downregulates FOXM1 activity

**Figure 2:**
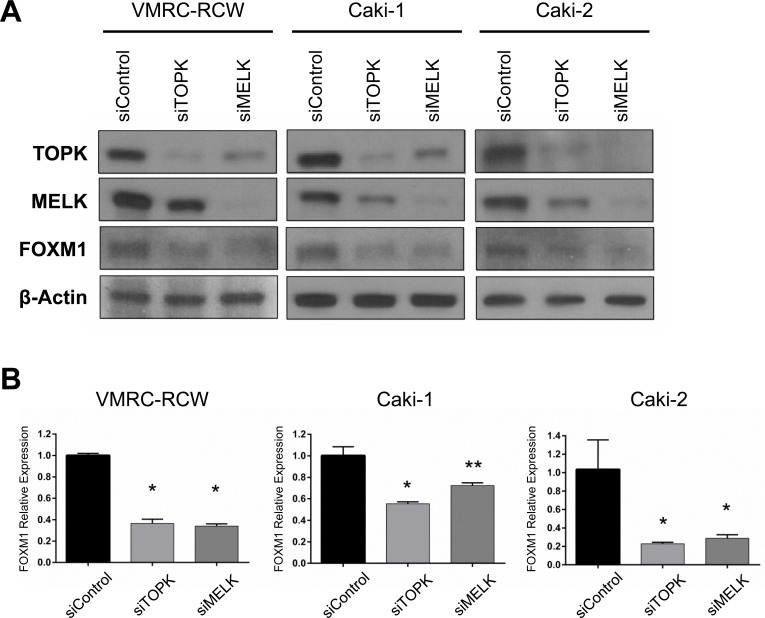
Both TOPK and MELK regulate expression of FOXM1 **A.** Silencing of TOPK expression downregulated FOXM1 protein level as similar as MELK affected on it in kidney cancer cell lines. **B.** Both siTOPK and siMELK downregulated *FOXM1* expression in the transcriptional level. *p < 0.01, **p < 0.05 compared with the corresponding value of the siControl group.

The transcriptional interaction between *TOPK* and *MELK* allowed us to examine any possible transcriptional factor that can influence on expression of these two genes. In the TCGA database, we found that *TOPK* and *MELK* expression levels were strongly correlated with that of *FOXM1* (Pearson's rank correlation is 0.73 and 0.82, respectively, Supplementary Figure S3, 4) [26, 27]. Moreover, we previously reported that the MELK inhibitor reduced expression of FOXM1 at protein level [22]. FOXM1 is a key transcriptional factor involved in the proliferation of cancer cells including leukemia cells that were very sensitive to both TOPK and MELK inhibitors [22, 28]. It is also notable that TOPK, MELK, and FOXM1 were suggested as cancer stem cell markers and listed in top 30 of the “consensus stemness ranking (CSR) signature” genes [29]. Based on these findings, we examined whether knockdown of TOPK or MELK could influence on the expression of FOXM1. As expected, FOXM1 protein level was decreased in kidney cancer cells transfected with siMELK (Figure 2A), probably due to the decrease of *FOXM1* transcription (Figure 2B). Intriguingly, siTOPK also resulted in the downregulation of FOXM1 not only in protein level but also in transcriptional level (Figure 2A and 2B).

### Influence of FOXM1 on *TOPK* and *MELK* expression

**Figure 3:**
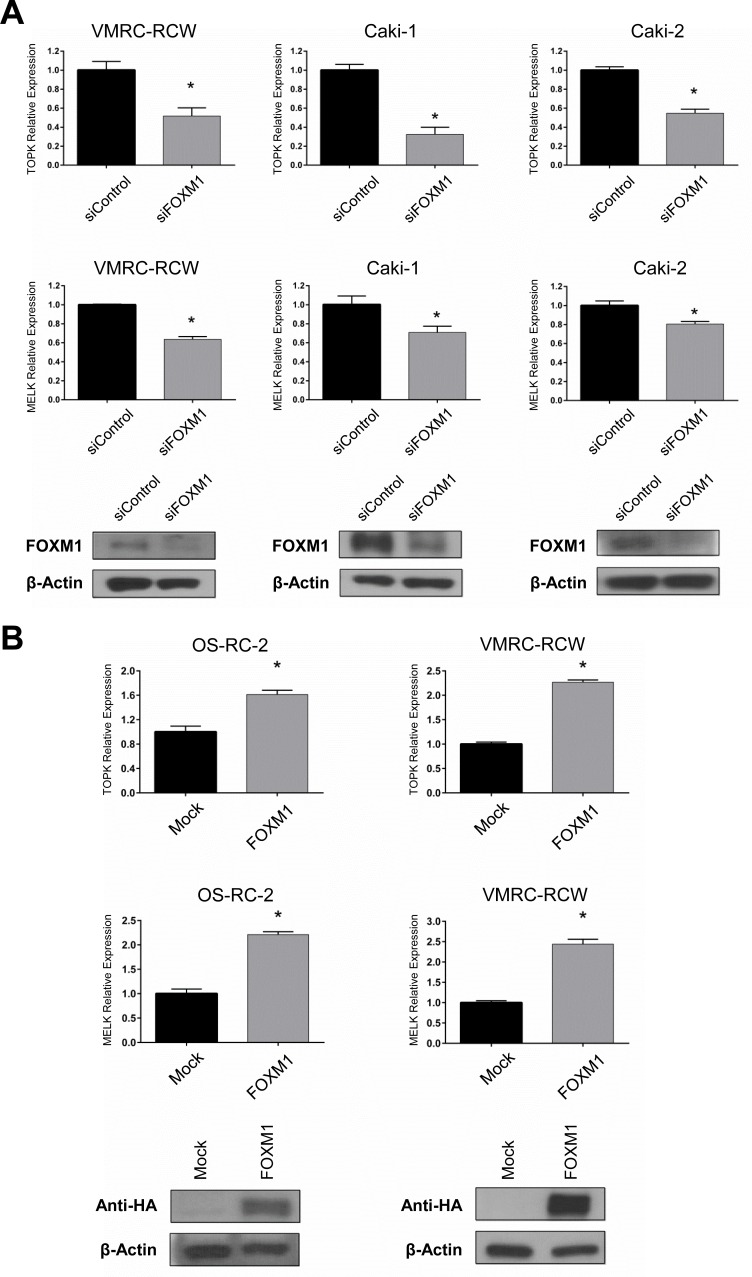
FOXM1 also regulates TOPK and MELK expression Expression level of *TOPK* and *MELK* were examined by RT-PCR after depletion or overexpression of FOXM1 in kidney cancer cells. **A.** FOXM1 knockdown led to the downregulation of *TOPK* and *MELK* expression in transcriptional levels. Western blots showed depletion of FOXM1 in kidney cancer cells 48 hrs after transfection with siFOXM1. **B.** The exogenous expression of FOXM1 was confirmed by western blot using anti-HA antibody, 48 hrs after transfection. The asterisk indicates *p* < 0.01 compared with the corresponding value of the siControl group.

Because FOXM1 was reported to bind promoter regions of *TOPK* and *MELK* genes in chromatin immunoprecipitation (ChIP) assay [30], we investigated transcriptional regulation on the *TOPK* and *MELK* genes by FOXM1. We first transfected kidney cancer cells with siFOXM1 and found that both *TOPK* and *MELK* mRNA levels were decreased in three kidney cancer cell lines compared with the cells treated with control siRNA (Figure 3A). We then exogenously introduced FOXM1 expression vector into two kidney cancer cell lines and found the significant elevation of *TOPK* and *MELK* mRNA expression in the cells transfected with FOXM1 expression vectors, while no change was observed in those transfected with the mock control vector (Figure 3B). Taken together, these results suggest that FOXM1 may function as a transcriptional factor that induces expression of *TOPK* and *MELK* genes in kidney cancer cells. Since either TOPK or MELK suppression with siRNA downregulated FOXM1, the feedback system among these three genes seems to be very complicated.

### Growth suppressive effect of OTS514 and OTS167 in kidney cancer cells

**Figure 4:**
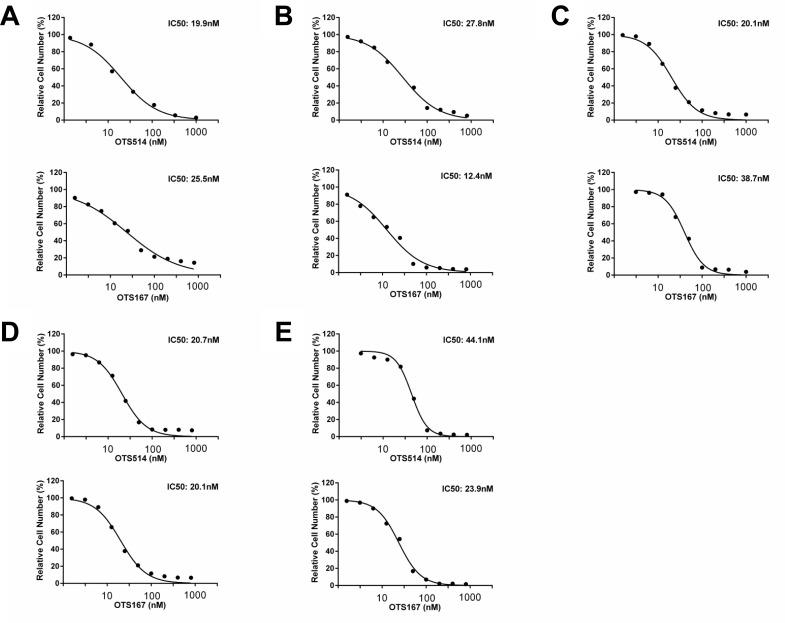
*In vitro* anti-proliferative effects of OTS514 and OTS167 in kidney cancer cell lines *In vitro* anti-proliferative effects of OTS514 and OTS167 in kidney cancer cell lines. Each graph shows growth inhibition curves of OTS514 and OTS167 for kidney cancer cell lines, VMRC-RCW **A.**, Caki-1 **B.**, Caki-2 **C.**, 769-P **D.**, and 786-O **E.**, in which both TOPK and MELK proteins are highly expressed.

Since both *TOPK* and *MELK* were overexpressed in kidney cancer [26, 27], we examined the half-maximum inhibitory concentration (IC_50_) value to measure the growth inhibitory effect of OTS514 and OTS167 on five kidney cancer cell lines, VMRC-RCW, Caki-1, Caki-2, 769-P and 786-O, in which TOPK and MELK were highly co-expressed (Figure 4A-4E). Our assay revealed low IC_50_ values as 19.9 to 44.1 nM for TOPK inhibitor OTS514 and 12.4 to 38.7 nM for MELK inhibitor OTS167.

### Downregulation of TOPK, MELK and FOXM1 by OTS514 and OTS167

**Figure 5:**
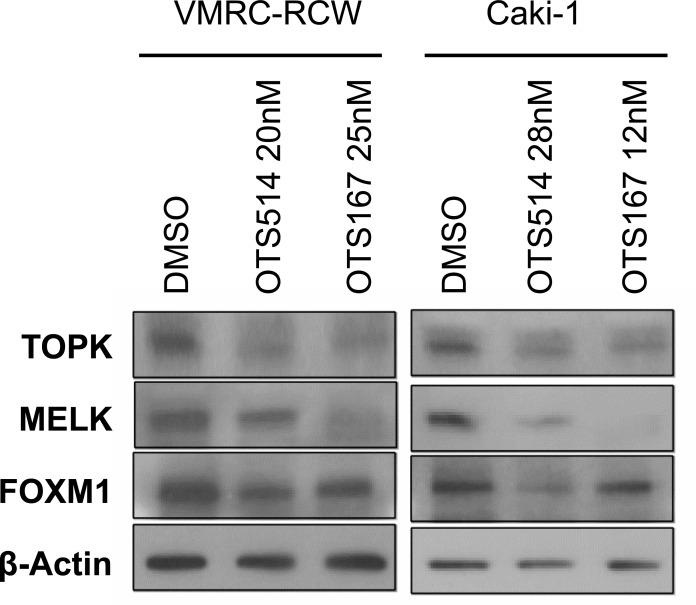
Downregulation of FOXM1 by OTS514 and OTS167 treatment Treatment with OTS514 or OTS167 at the concentration of their IC_50_ values reduced FOXM1 protein level in VMRC-RCW and Caki-1 cells, as examined by western blot analysis.

Our previous study in leukemic cells showed that downregulation of MELK resulted in the suppression of FOXM1 [22]. Similarly, our results in kidney cancer cells treated with the IC_50_ concentration of MELK inhibitor (OTS167) showed decrease of MELK and TOPK proteins as well as FOXM1 protein (Figure 5). This downregulation effect of three proteins by OTS167 was in concordant with that of siMELK treatment (Figure 2A). Interestingly, treatment with TOPK inhibitor (OTS514) at the IC_50_ condition also remarkably reduced the protein level of all three proteins (Figure 5), similar to the results obtained by siTOPK treatment (Figure 2A). Collectively, our assays implied that both inhibitors are very effective to suppress kidney cancer cell growth through reduction of TOPK, MELK, and FOXM1 proteins that may cooperatively constitute a critical signal pathway in kidney cancer cells.

### Dual TOPK and MELK inhibition additively reduced cell viability and increased apoptosis

**Figure 6:**
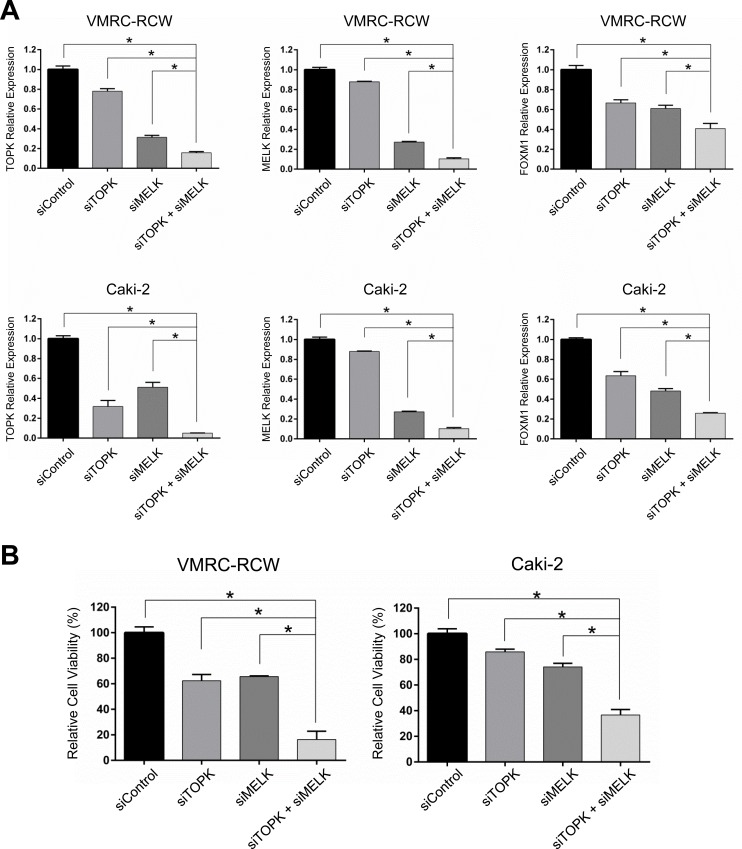
Additive effects with dual TOPK and MELK inhibition with low-dose siRNAs in kidney cancer cells **A.** Dual knockdown with low-dose siTOPK (50 pmol) + siMELK (50pmol) additively suppressed *TOPK*, *MELK* and *FOXM1* expression in transcriptional level compared with either knockdown of TOPK or MELK alone. **B.** Dual knockdown of TOPK and MELK with low-dose siRNAs showed drastic decrease in the cell viability compared with either knockdown of TOPK or MELK alone. The asterisk indicates *p* < 0.01 compared with the corresponding value of the siControl group.

**Figure 7:**
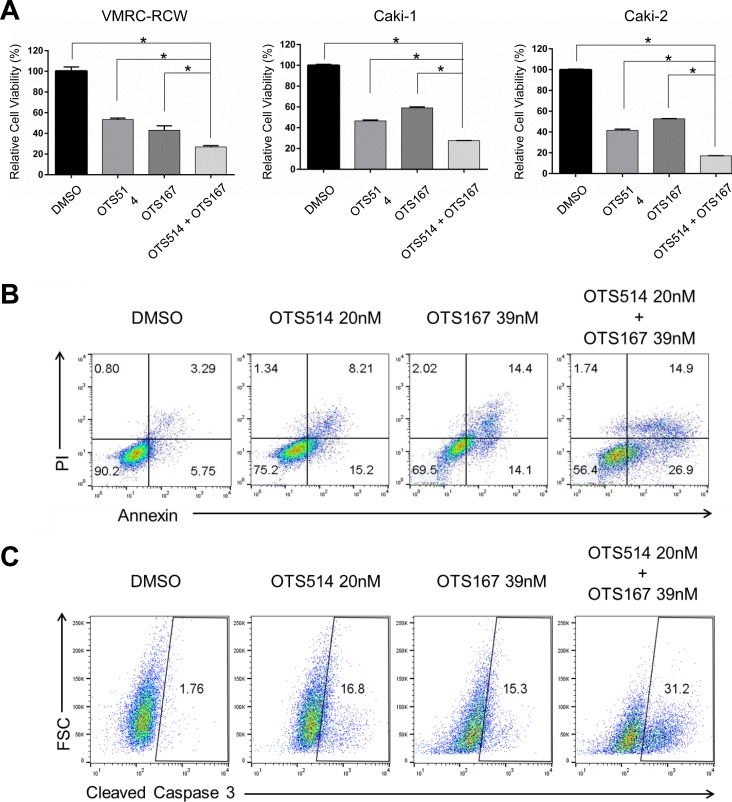
The effects in the decrease of kidney cancer cell viability by combination of OTS514 and OTS167 **A.** The combination of OTS514 and OTS167 at the concentration of IC_50_ values significantly revealed growth-suppressive effects on kidney cancer cells compared with the treatment of a single compound. The asterisk indicates *p* < 0.01 compared with the treatment of a single compound. **B.** Early stage of apoptosis assessed by Annexin and PI staining of kidney cancer cells. The combination of OTS514 and OTS167 at the concentration of IC_50_ values significantly increased 14.7 ± 2.6 % (compared with OTS514 alone) or 10.6 ± 2.6 % (compared with OTS167 alone) of apoptotic cells (*p* = 0.001 and *p* = 0.001, respectively). **C.** Apoptosis was examined by the levels of cleaved caspase 3 in kidney cancer cells. Dual inhibition with OTS514 and OTS167 significantly increased 10.8 ± 3.1 % (compared with OTS514 alone) or 12.5 ± 2.9 % (compared with OTS167 alone) of late stage population of apoptosis compared with either inhibition alone (*p* = 0.03 and *p* = 0.02, respectively). Flow cytometry figures are representative of three independent experiments.

Although a detailed molecular mechanism in regulation of TOPK, MELK, and FOXM1 needs to be clarified, our findings from siRNA experiments and assays using small molecule inhibitors strongly indicated some kind of feedback loop machinery among the three molecules. Taking into consideration of clinical application, we evaluated a combinatory inhibitory effect against TOPK and MELK. We transfected the low amount of siRNA (50 pmol each) that is equivalent to one-fourth of siRNA that we used knockdown assays shown in Figure 1. Although this amount of siRNA was capable to reduce *TOPK* and *MELK* expression modestly, combination of siTOPK and siMELK at this concentration resulted in much stronger effect on reduction of *TOPK* and *MELK* expression as well as *FOXM1* expression (Figure 6A). Concordantly, combination of siTOPK and siMELK effectively achieved decrease of cell viability of kidney cancer cells (Figure 6B). Subsequently, three kidney cancer cell lines were treated with IC_50_ values of OTS514 or/and OTS167. As expected, MTT assay revealed that the combination treatment of OTS514 and OTS167 significantly decreased cell viability for all of the kidney cancer cells examined, compared with the treatment with a single compound (Figure 7A). In addition, we assessed apoptosis of cancer cells (Caki-2) by flow cytometry and observed significantly increased apoptosis of 14.7 ± 2.6 % (*p* = 0.001, for OTS514) or 10.6 ± 2.6 % (*p* = 0.001, for OTS167) at an early time-point of drug treatment (Annexin-positive but PI-negative field) when cells were treated with the two drugs (Figure 7B). Consistent with these findings, the cleaved caspase 3 was significantly increased in the cells with the combination of OTS514 and OTS167 (*p* = 0.03 and *p* = 0.02, respectively, Figure 7C). Effective induction of apoptosis by combination of two compounds was also observed in kidney cancer VMRC-RCW cells (Supplementary Figure S5).

## DISCUSSION

Despite advances in the novel treatment modalities, 5-year cancer specific survival rate of kidney cancer patients who are at the advanced stage still remains around 10% [1, 2, 4]. This low rate may be partially explained by the poor response of kidney cancer to chemotherapy and radiation therapy. Moreover, patients who once relapsed or are at stage IV have limited therapeutic options such as kinase inhibitors and mTOR inhibitors, which often cause severe adverse effects [5, 6]. Given the limitation of these therapeutic choices, novel effective anti-cancer drugs should be developed urgently.

According to the Oncomine database, *TOPK* and *MELK* are upregulated in kidney cancer and considered as promising molecular targets because of their cancer-restricted expression patterns [26, 27]. Previous studies have shown that MELK inhibition effectively reduced expression of an oncogenic transcriptional factor, FOXM1 [22]. In the present study, we further demonstrated that TOPK knockdown also caused suppression of FOXM1 expression as similar to MELK knockdown. We also revealed that the growth of kidney cancer was suppressed by either TOPK or MELK inhibition.

The transcriptional interaction among TOPK, MELK and FOXM1 seems to be very complicated. We have demonstrated that treatment of cancer cells with either siTOPK or siMELK downregulated FOXM1 both in protein level and in transcriptional level. These results implied the presence of some feedback mechanism that regulates expression of the *FOXM1* gene. To elucidate this complex feedback system, we conducted large-scale gene network analysis by the SiGN-BN algorithm using a supercomputer system. However, we could not find any possible candidate factors involved in the MELK-TOPK-FOXM1 pathway in any deposited cancer database. This may be partially explained by (1) lack of relevant kidney cancer dataset in the database, (2) indirect regulation of these three genes in the feedback loop including multiple mediator genes, or (3) both. Although further molecular mechanism should be elucidated, our findings suggested the existence of a complex feedback loop among TOPK, MELK and FOXM1. Because knockdown of TOPK and MELK revealed much stronger effect on the growth suppression of kidney cancer cells than that of FOXM1 (data not shown), it is almost certain that TOPK and MELK are more attractive molecular targets than FOXM1 to inhibit signaling pathways essential for cancer proliferation, although it remains unclear which will serve as an upstream of others and play more fundamental roles in kidney cancer.

Pre-clinical studies showed that treatment with TOPK inhibitor (OTS964, a derivative of OTS514) could induce complete regression of tumors but led to adverse reactions in hematopoietic cells [23]. On the other hand, MELK inhibitor (OTS167) did not cause any adverse reactions at the effective dose although it did not result in complete regression of tumors in mice model [24]. In fact, we demonstrated that dual blockade with low doses of TOPK inhibitor (OTS514) and MELK inhibitor (OTS167) achieved the additive cancer cell killing effects rather compared with the single treatment. Considering advantages and disadvantages of these compounds, it will be essential to figure out the most effective combination dose to achieve complete tumor elimination with minimum risk of adverse reaction.

In summary, we suggest that both TOPK and MELK are attractive molecular targets for kidney cancer treatment and they constitute a feedback loop with an oncogenic transcriptional factor FOXM1. Given that the beneficial effects of dual administration of low dose OTS514 and OTS167, this combination strategy will provide more potential cancer therapeutics that could be applied to a various types of human malignancy.

## MATERIALS AND METHODS

### Cell lines, plasmids, oligo siRNAs and transfection

769-P, 786-O, A-498, A-704, Caki-1, Caki-2 and ACHN cells were purchased from the American Type Culture Collection (ATCC; Manassas, VA). OS-RC-2, TUHR-10TKB and TUHR-14TKB cells were provided by RIKEN BioResource Center (Tsukuba, Japan). VMRC-RCW was provided by the Cell Resource Center for Biomedical Research, Institute of Development, Aging and Cancer, Tohoku University. SNU228, SNU267, SNU333, SNU349 and SNU482 were provided by Dr. Jae-Gahb Park (Korean Cell Line Bank, Seoul, Korea). All cells were cultured under appropriate media recommended by suppliers with 10% FBS and 1% antibiotic-antimycotic solution (Sigma-Aldrich, St. Louis, MO). All cells were maintained at 37°C in humidified air with 5% CO_2_. For knockdown experiments, cells were transfected with 200 pmol or 50 pmol (low dose experiment) of oligo siRNA using Lipofectamine RNAiMAX (Invitrogen, Carlsbad, CA) according to manufacturer's instructions. The target sequences of oligo siRNAs were as follows: 5′-GUGUGGCUUGCGUAAAUAA-3′ for TOPK; 5′-GACAUCCUAUCUAGCUGCA-3′ for MELK; and 5′-GGACCACUUUCCCUACUU-3′ for FOXM1. To construct vectors designed to express FOXM1 (NM_021953.3), the entire coding sequences were amplified by RT-PCR and cloned into the pCAGGSnHc expression vector. Plasmids were transfected using FugeneHD (Roche, Basel, Switzerland) according to the supplier's recommendations.

### Western blot analysis and antibodies

Western blot analysis was performed by normalization to β-actin or the baseline expression level. Cells were lysed with IP lysis buffer (Thermo Scientific, Waltham, MA) containing protease inhibitor cocktail III (Millipore, Billerica, MA). The proteins were separated by electrophoresis using 4-20% or 7.5% SDS-PAGE gel, and transferred onto nitrocellulose membrane. The membranes were incubated with the first antibody, respectively: anti-TOPK antibody (BD Biosciences, San Jose, CA), anti-FOXM1 antibody (Santa Cruz Biotechnology, Santa Cruz, CA), anti-HA (Roche), or anti-β-actin (Sigma-Aldrich). Finally, the membrane was incubated with horseradish peroxidase-conjugated secondary antibody and protein bands were visualized by enhanced chemiluminescence detection reagents (GE Healthcare, Pittsburgh, PA). We generated mouse anti-MELK monoclonal antibodies using partial recombinant MELK protein (264-601 amino acids of MELK) as an immunogen by the methods as described previously [31].

### Cell viability assay

For methyl thiazolyl tetrazolium (MTT) assay, cancer cells were seeded into 24-well flat-bottom plates (BD Falcon) at 5× 10^4^ cells per well, and mixed with oligo siRNA or TOPK and/or MELK inhibitors. Compounds of OTS514 and OTS167 were kindly provided by OncoTherapy Science Inc. (Kawasaki, Japan). Cells were cultured at 37°C under 5% CO_2_ for 72 h. The Cell counting kit-8 (Dojindo Molecular Technologies, Inc., Kumamoto, Japan) was used for MTT reaction and examined the cell viability. After reaction for 1 to 3 hr, 100 μL of supernatant was transferred into a 96-well plate and read in a microplate reader at 450 nm. For the viability and apoptosis analyses, cells were collected, spun down then washed with PBS and resuspended in 50 μL of binding buffer containing 2 μL of Annexin V (eBioscience, San Diego, CA). After 20 min incubation, cells were stained with 100 μL of binding buffer containing 1 μL propidium iodide (PI) (eBioscience). For a cleaved caspase 3 assay, cells were collected, spun down, then washed with PBS, resuspended in 500 μL Cytofix/Cytoperm solution (eBioscience) and incubated cells on ice for 20 min. Subsequently, cells were spun down, washed with Perm/Wash buffer (eBioscience) and resuspended with 100uL of buffer containing 20 μL of cleaved caspase 3 antibody (eBioscience). Fluorescence was quantified by flow cytometry (FACS LSRII; Becton Dickinson, San Jose, CA). Flow Jo software (Treestar, Ashland, OR) was used to identify Annexin and PI positive cell subpopulations and cleaved caspase 3- positive cell subpopulations.

### Real-time RT-PCR

Total RNA was extracted from cell monolayers using RNeasy Mini Kit (Qiagen, Valencia, CA) according to the manufacturer's directions. Total RNA (1 - 2 μg) was reversely transcribed using SuperScript III First-Strand Synthesis System (Invitrogen) following the manufacturer's instructions. Aliquots of the reverse transcription product were quantified by real-time RT-PCR. The RT-PCR was performed using primers listed below using the ViiA 7 system (Life Technologies, Grand Island, NY). The expression levels were normalized with that of *GAPDH*. The PCR primer sequences were 5′-AGACCCTAAAGATCGTCCTTCTG-3′ and 5′-GTGTTTTAAGTCAGCATGAGCAG-3′ for *TOPK*; 5′-GCTGCAAGGTATAATTGATGGA-3′ and 5′-CAGTAACATAATGACAGATGGGC-3′ for *MELK*; 5′-CAACCGCTACTTGACATTGGA-3′ and 5′-TCACCGGGAACTGGATAGG-3′ for *FOXM1* and 5′-CGACCACTTTGTCAAGCTCA-3′ and 5′-GGTTGAGCACAGGGTACTTTATT-3′ for *GAPDH*.

### Gene network analysis

For the gene network analysis, gene expression datasets deposited in the NCBI GEO were analyzed by SiGN-BN software with supercomputer “K computer” in Advanced Institute for Computational Science Riken, Japan [32, 33]. The resulting networks were compiled as a gene network databases, The Cancer Network Galaxy (TCNG, http://tcng.hgc.jp/). We searched for sum-networks containing three genes, *TOPK*, *MELK* and *FOXM1* and their first and second neighbors.

### Statistical analysis

Data were expressed as mean ± one standard deviation. Differences between two groups were examined for significance using student's *t* test. Differences were considered significant at *p* < 0.05. Experiments were performed in triplicate.

## SUPPLEMENTARY MATERIAL FIGURES


