# Supplementary material for: Oncogenic roles of TOPK and MELK, and effective growth suppression by small molecular inhibitors in kidney cancer cells
Source: Oncotarget. 2016 Feb 26;7(14):17652–64. doi: 10.18632/oncotarget.7755 (PMC4951240; doi:10.18632/oncotarget.7755)
Supplement: Supplementary file 1 [file oncotarget-07-17652-s001.pdf]

# Oncogenic roles of TOPK and MELK, and effective growth suppression by small molecular inhibitors in kidney cancer cells

## Supplementary Material

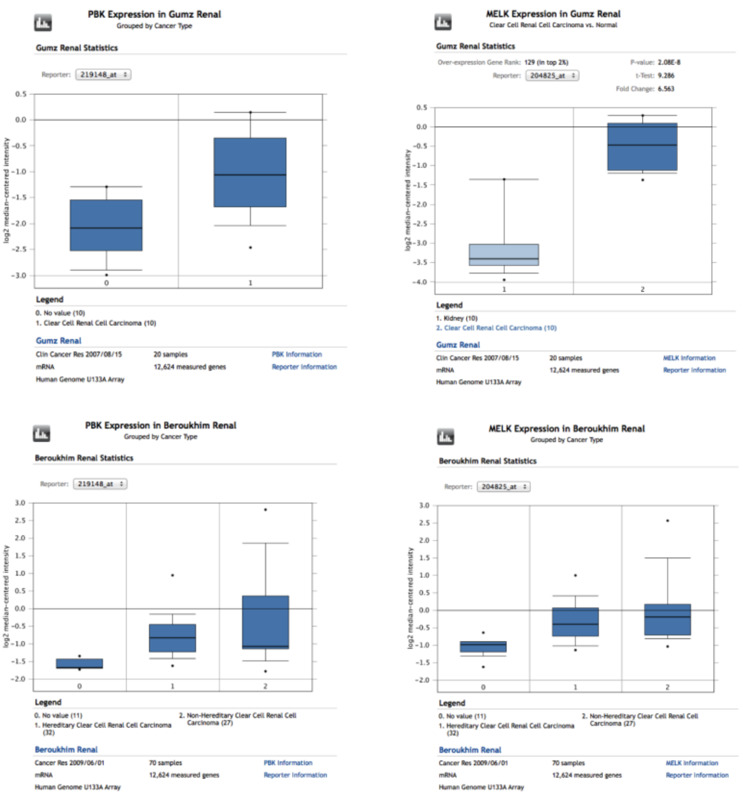


**Supplementary Figure 1.** Oncomine database shows high expression of *TOPK (PBK)* and *MELK* in kidney cancer compared with normal kidney tissues.

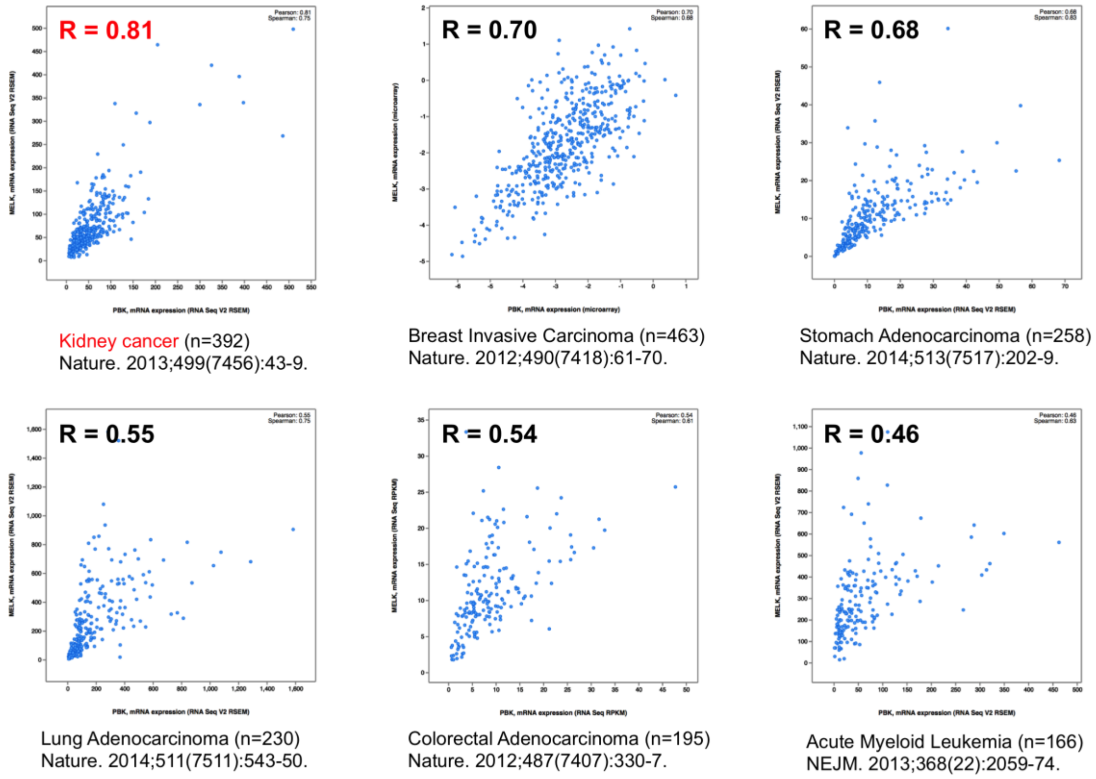


**Supplementary Figure 2.** Correlation between *TOPK* and *MELK* mRNA expression in kidney cancer patients. Pearson's rank correlation is 0.81.

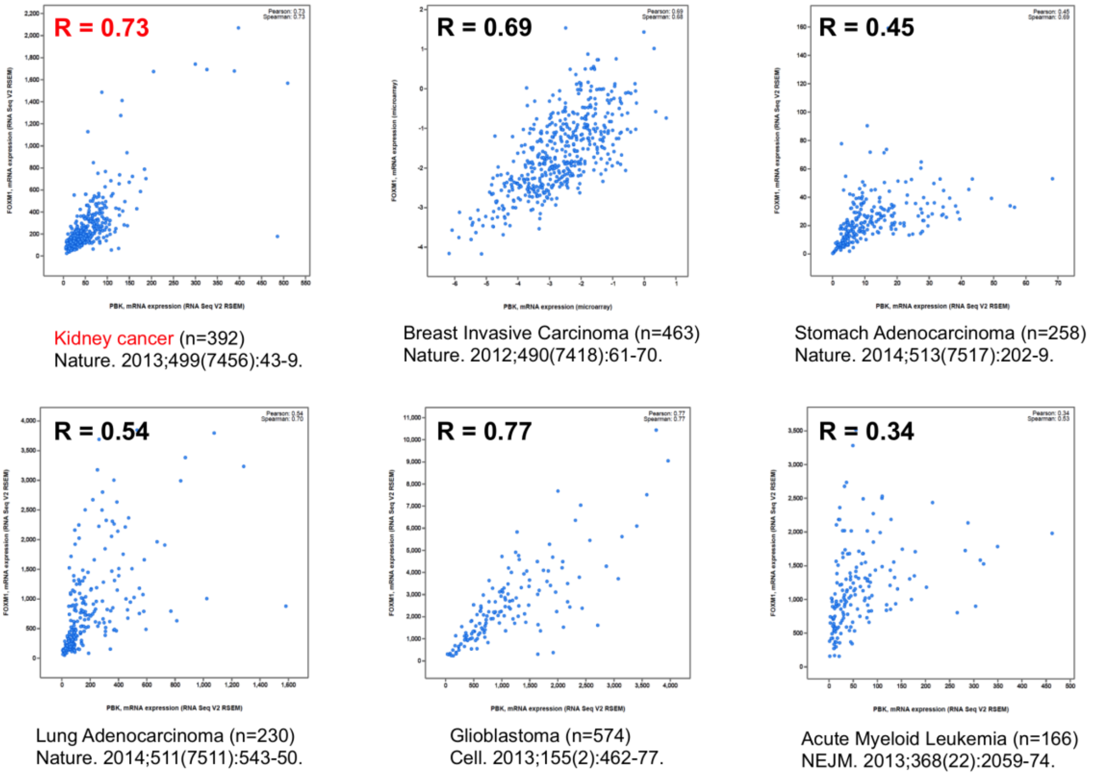


**Supplementary Figure 3.** Correlation between *TOPK* and *FOXM1* mRNA expression in kidney cancer patients. Pearson's rank correlation is 0.73.

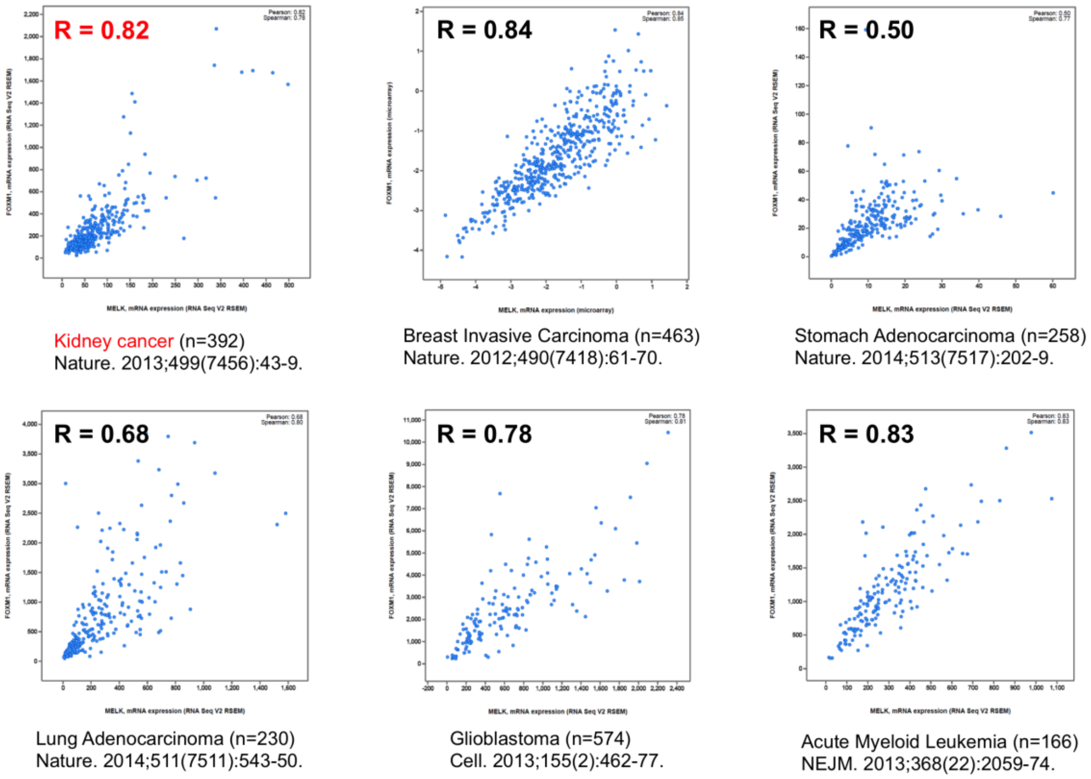


**Supplementary Figure 4.** Correlation between *MELK* and *FOXM1* mRNA expression in kidney cancer patients. Pearson’s rank correlation is 0.82.

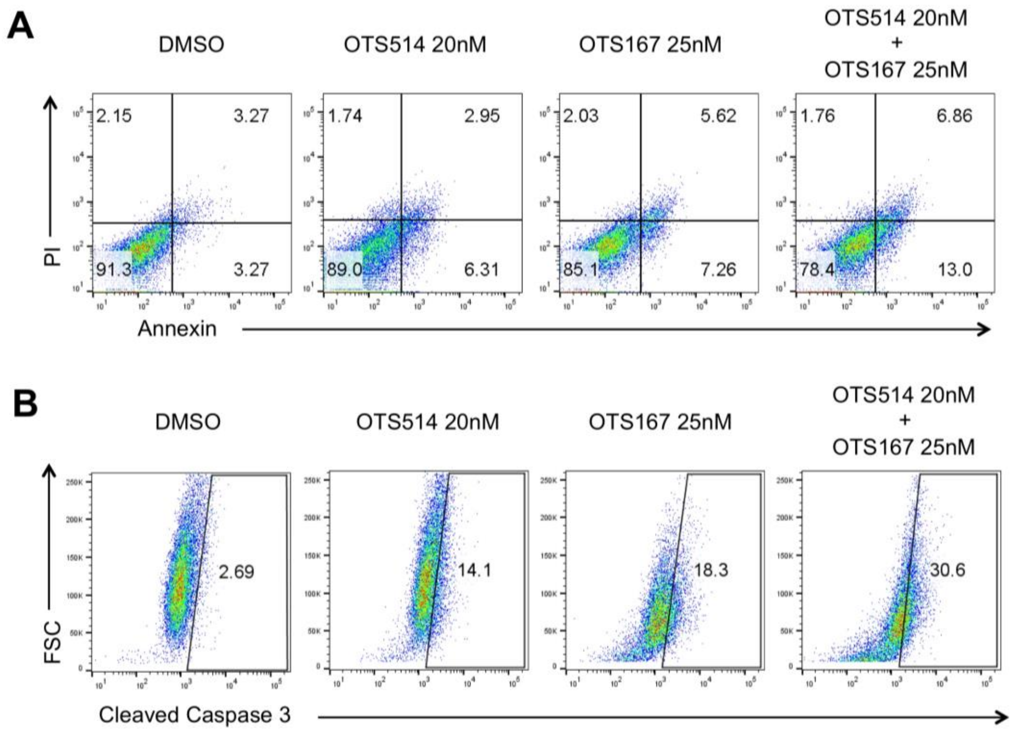


**Supplementary Figure 5.** Additive growth inhibitory effect by a combination of OTS514 and OTS167 in VMRC-RCW cells. (**A**) Combinational treatment of OTS514 and OTS167 significantly increased 7.5 ± 1.1 % (compared with OTS514 alone) or 7.2 ± 1.8 % (compared with OTS167 alone) of apoptotic cells at an early time-point of drug treatment ($p = 0.001$ and $p = 0.001$, respectively). (**B**) Another cell apoptosis marker, cleaved caspase 3, was also significantly increased in the cells treated with combination of OTS514 and OTS167, compared with monotherapy ($p = 0.02$ and $p = 0.04$, respectively).
